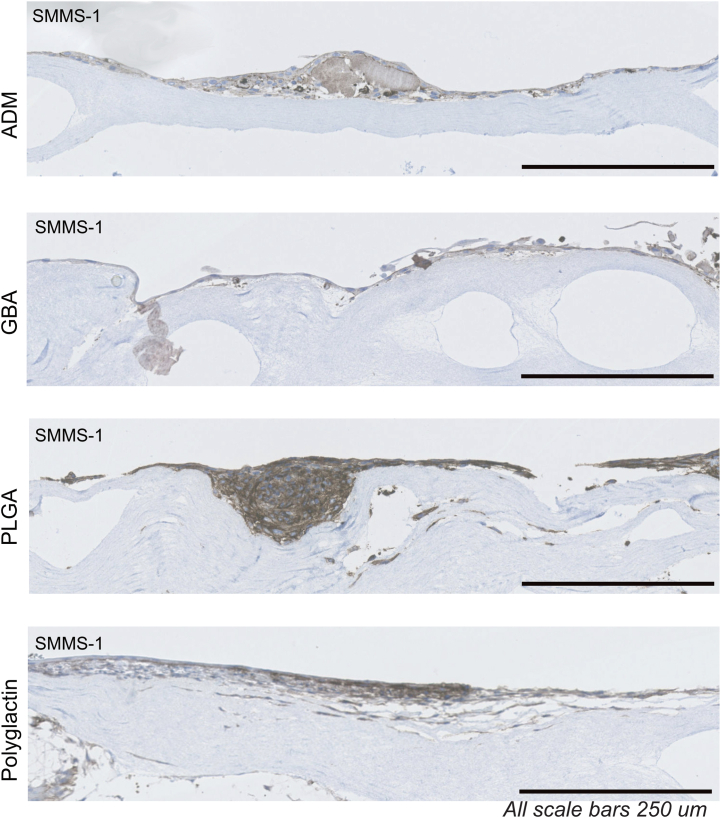# A perioperative layered autologous tissue expansion graft for hollow organ repair

**DOI:** 10.1016/j.heliyon.2024.e25275

**Published:** 2024-01-24

**Authors:** Oliver Willacy, Nikolai Juul, Loai Taouzlak, Clara I. Chamorro, Fatemeh Ajallouiean, Magdalena Fossum

**Affiliations:** a Laboratory of Tissue Engineering, Rigshospitalet, Faculty of Health and Medical Sciences, University of Copenhagen, Copenhagen, Denmark; b Division of Pediatric Surgery, Department of Surgery and Transplantation, Copenhagen University Hospital Rigshospitalet, Copenhagen, Denmark; c Department of Health Technology, Technical University of Denmark, 2800: Kgs, Lyngby, Denmark; d The Danish National Research Foundation and Villum Foundation's Center for Intelligent Drug Delivery and Sensing Using Microcontainers and Nanomechanics (IDUN), Department of Health Technology, Technical University of Denmark, Ørsted Plads, 2800: Kgs, Lyngby, Denmark; e Laboratory of Tissue Engineering, Department of Women's and Children's Health, Karolinska Institutet, Stockholm, Sweden

**Keywords:** Biomaterials, Hydrogel, Translational medicine, Tissue engineering, Tissue expansion, PLATE graft

## Abstract

Tissue engineering has not been widely adopted in clinical settings for several reasons, including technical challenges, high costs, and regulatory complexity. Here, we introduce the Perioperative Layered Autologous Tissue Expansion graft (PLATE graft), a composite biomaterial and collagen-reinforced construct with autologous epithelium on one side and smooth muscle tissue on the other. Designed to mimic the structure and function of natural hollow organs, the PLATE graft is unique in that it can be produced in a standard operating theatre and is cost-effective. In this proof-of-principle study, we test its regenerative performance in eight different organs, present biomechanical and permeability tests, and finally explore its *in vivo* performance in live rabbits.

## Graphical abstract

**Figure:**
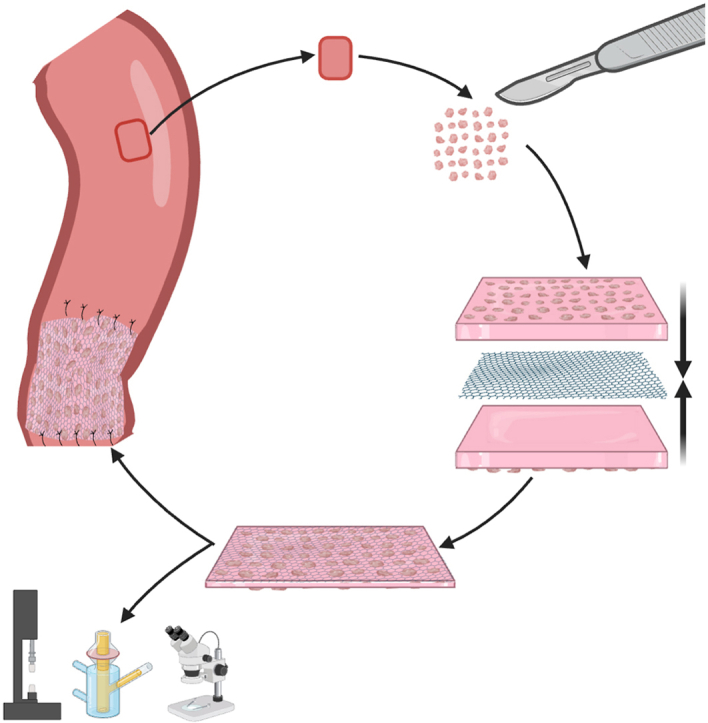

## 1 Introduction

Birth defects are detected in 3–5 % of newborns and represent a spectrum of congenital malformations that can cause severe health and developmental complications [1]. Most common are the cardiac anomalies, however, hollow organs are often involved, as defects can occur anywhere along the gastro-intestinal canal, respiratory tract, urinary system, reproductive organs and in vascular structures. Diseased hollow organs also occur in adult patients after cancer treatments, traumas or in association with other acquired conditions. The surgical repair often involves the removal of the pathological region and reconnecting the loose ends of the organ in one or more anastomoses. However, the success of this procedure can be hindered by a shortage of healthy grafting tissue, which makes it challenging to reconnect the ends. Depending on the location of the affected organ, failure to restore its function can lead to significant morbidity and even mortality [2,3].

Hollow organs from the gastrointestinal and genitourinary tract share a similar overall histological composition with a luminal epithelium covering layers of connective- and muscular tissues [4]. These organs are less complex to engineer than solid organs, possibly due to better vascularization, reduced thickness that enables passive diffusion, and the better availability of biomimetic scaffolds [5]. Thus, the creation of functional autologous hollow organ tissue grafts using *ex vivo* culture techniques, has been expected to revolutionise reconstructive surgery [6]. Indeed, over the last decades, many first-in-man clinical trials have been performed using tissue-cultured autologous grafts including epidermis [7], cartilage [8], pulmonary artery [9], urethra [10], bladder [11], trachea [12], vagina [13], and oesophagus [14]. However, these new methods have seldom been implemented in broad clinical settings, remaining largely academic achievements rather than available treatment options [15]. One of the key elements hindering clinical translation of the conventional methods for tissue engineering is the need for resource-intensive good manufacturing practice (GMP) facilities to culture engineered organs in bioreactors *ex vivo*. This regulatory framework was implemented in 2001 by the Food and Drug Administration (FDA) and later in 2007 by the European Medicines Agency (EMA) as an important step for patient safety. Unfortunately, the implementation has proven to be a hurdle for funding and clinical implementation of new tissue engineering technologies [16,17]. Moreover, the traditional tissue engineering methods, whereby autologous cells are seeded onto a biomaterial, require multiple surgical steps from initial biopsy (for cell isolation and *ex vivo* cultivation) to re-implantation of the tissue-cultured organ back into the patient. The additional procedure often delays surgical reconstruction by several months, which is inconvenient for patients in urgent need [10].

The Perioperative Layered Autologous Tissue Expansion graft (PLATE graft), introduced in this paper, meets three key criteria for enhanced clinical potential: 1) not using ex-vivo tissue culture and instead inserting the grafts directly into the final tissue of interest using the body as a bioreactor for autologous tissue regeneration and expansion, 2) it utilizes low-cost materials and kits, and 3) it can be completed in a single-stage surgical procedure. Central to this approach was the identification of a resorbable biomaterial that could mimic the organs' biomechanical properties while providing a scaffold for two layers of collagen and autologous tissue, and surgical handling. We therefore tested and compared four different biomaterials. Our underlying hypothesis is that the biomaterial provides short-term biomechanical support, while the autologous minced tissue particles, referred to as micrografts, furnish the substrate for long-term cellularization. If proven effective, PLATE grafts could democratise access to tissue engineering technologies worldwide, including in countries with limited healthcare resources.

This study aims to explore the graft's performance concerning regeneration, biomechanics, and permeability and to compare it to various native organs, focusing specifically on potential applications in the vagina.

## 2 Results

### 2.1 Graft design and biomaterial comparisons

**Fig. 1:**
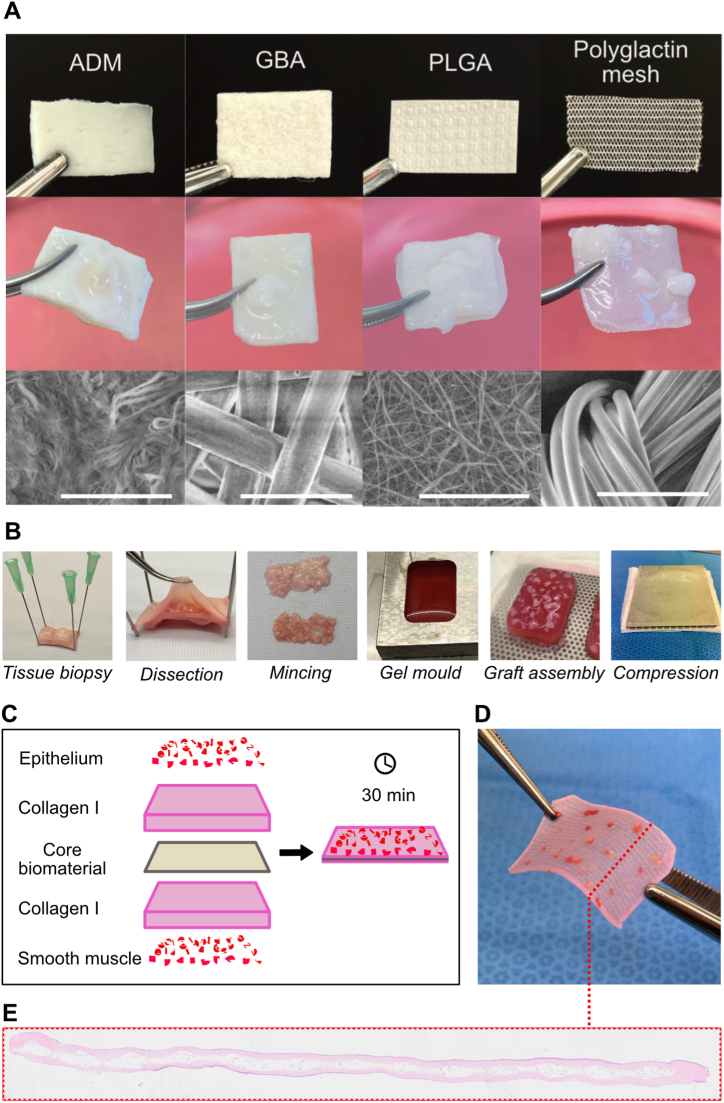
PLATE graft methodology and biomaterial overview. (A) Macroscopic images of the four biomaterials before (top) and after graft fabrication and 4 weeks of tissue culture (middle). Ultrastructural representation of the biomaterials off the shelf (bottom). ADM = Acellular dermal matrix, GBA = Gore BIO-A, PLGA = poly(lactic-co-glycolide). Scale bars 100 μm. **(B)** The steps to make a PLATE graft: Epithelium and smooth muscle layers were dissected and minced. Collagen gel was cast in a mould with the biomaterial inside, and the micrografts were added to either side of the construct before compression. **(C)** Schematic illustration of the five layers that are compressed together to form a graft. **(D)** Final appearance of a polyglactin mesh graft after compression. **(E)** Full-length cross-section histology of a polyglactin mesh graft after 3 weeks of tissue culture, H&E.

Four different resorbable biomaterials were tested for compatibility with the method including: biological acellular dermal matrix (referred to as ADM), GORE ® BIO A® with 67 % polyglycolic acid and 33 % trimethylene carbonate (referred to as GBA), a custom-made biomaterial: poly(lactic-co-glycolide) (referred to as PLGA) and finally, polyglactin 910 mesh (knitted Vicryl® Mesh). These four biomaterials were selected to encompass both biological and synthetic materials with differing resorption times, thicknesses, and surface patterns. Macroscopically, the ADM and GBA were thicker than the thin sheet of PLGA or the knitted polyglactin mesh. Ultrastructurally, ADM and PLGA had more tightly woven fibers than the more loosely organized GBA and polyglactin mesh (Fig. 1A). Grafts could be created in 30 min from a small autologous tissue biopsy that was dissected, minced, distributed, and compressed into a composite construct of both collagen gel and a biomaterial (Fig. 1B&C). Once compressed, the grafts were ready for immediate use inside the host organism or for *in vitro* culture and analysis (Fig. 1D&E).

### 2.2 Biomechanical performance and permeability

**Fig. 2:**
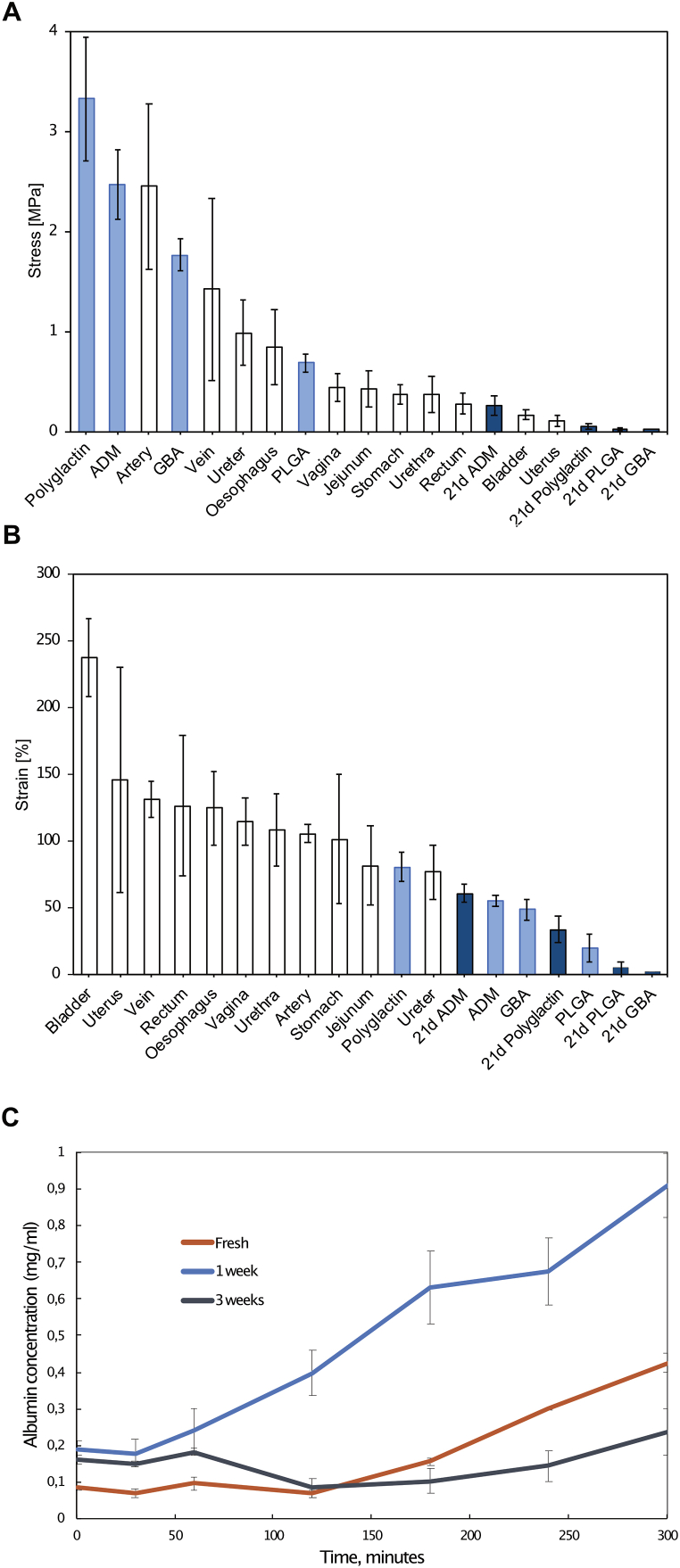
Biomechanics and permeability. A Bar charts representing stress values during ultimate tensile strength assessment of 11 fresh porcine organs (n = 9 pr condition), engineered grafts made from various biomaterials after 0 days of culture (light blue, n = 3 pr condition), or after 21 days of tissue culture (dark blue, n = 3 pr condition). Error bars = SD. **(B)** Bar charts representing strain values during ultimate tensile strength assessment of 11 fresh porcine organs (n = 9 pr condition), engineered grafts made from various biomaterials after 0 days of culture (light blue, n = 3 pr condition), or after 21 days of tissue culture (dark blue, n = 3 pr condition). Error bars = SD. **(C)** Albumin permeability through vaginal PLATE graft, when used as a membrane to separate two chambers, comparing polyglactin grafts fresh (orange), after 1 week of tissue culture (light blue) or after three weeks of tissue culture (dark blue). Error bars = SEM. (For interpretation of the references to colour in this figure legend, the reader is referred to the Web version of this article.)

To place the biomaterials within a biological context, tensile tests measuring the ultimate tensile strength and strain levels were performed and compared to 11 different reference porcine organs. The urinary bladder stood out, able to withstand 238 ± 29 % strain before rupture, the highest measure detected among all biological or engineered conditions. Vascular structures (artery and vein) were the strongest biological organs tested but were surpassed by several biomaterial-reinforced grafts (Fig. 2A&B).

The polyglactin mesh had the highest ultimate tensile strength of 3.32 ± 0.62 MPa, withstanding significantly more stress and strain than both GBA and PLGA (p < 0.05). At 80.5 %, polyglactin mesh was also significantly more flexible than any other graft type tested (p < 0.05). However, it was still less flexible than most of the reference organs. To assess how graft maturation affected biomechanical performance over time, tensile tests were performed on grafts incubated for three weeks. GBA had lost its structural integrity, making tensile tests impossible at this time point. Polyglactin mesh, ADM, and PLGA all tolerated significantly less stress after 21 days of culture (p < 0.005). Polyglactin mesh also underwent a significant drop in strain (33.24 ± 10.01 %, p = 0.005). The polyglactin mesh grafts were further assessed with permeability assays that quantified the diffusion of albumin particles over the graft constructs over time. Grafts that had been cultured for one week were significantly more permeable than grafts that had been cultured for three weeks (0.91 mg. albumin/hr/cm^2^ VS 0.24 mg albumin/hr/cm^2^, p < 0.05). There was no significant difference in permeability between freshly compressed grafts and grafts that had been cultured for three weeks: (0.43 mg. abumin/hr/cm^2^ VS 0.24 mg albumin/hr/cm^2^, p = 0.19) (Fig. 2C).

### 2.3 Vaginal tissue regeneration *in vitro*

**Fig. 3:**
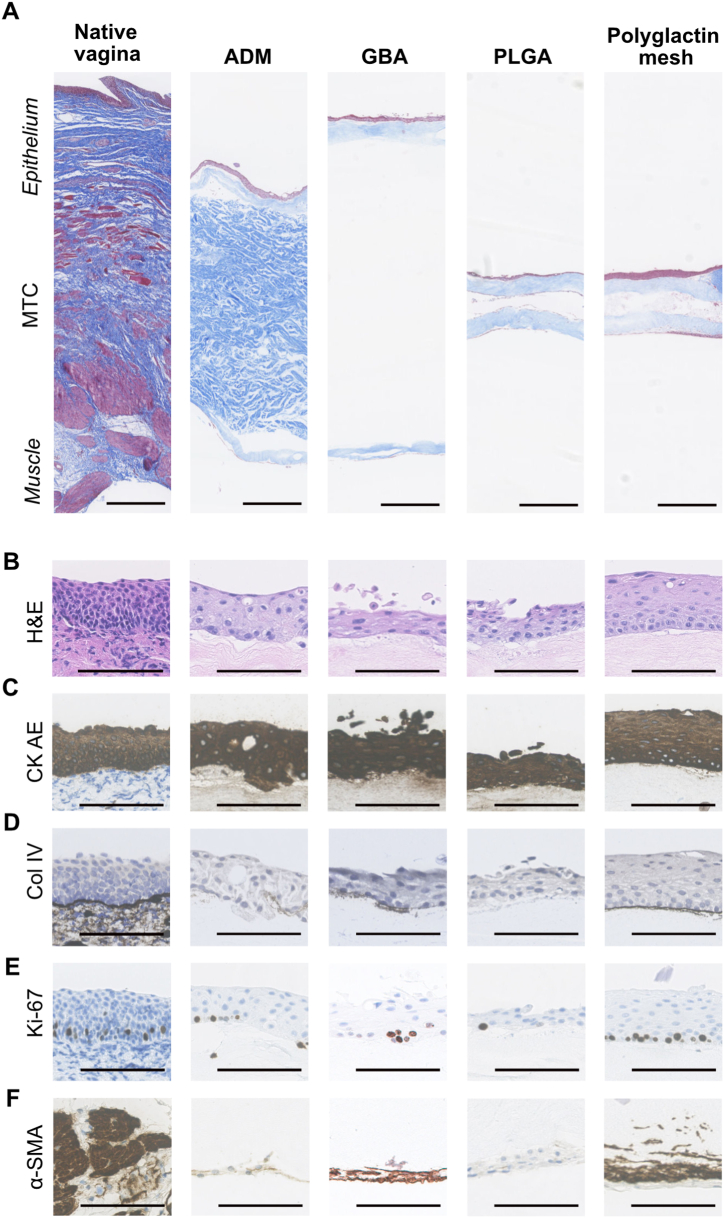
Histological evaluation of PLATE grafts made from porcine vaginal tissue. (A) Full-thickness cross-section of the native porcine vagina and grafts made with the four different biomaterials after four weeks of culture. MTC = Masson's trichrome, scale bar: 500 μm. **(B)** Vaginal mucosa morphology analysed with H&E. **(C)** CK-AE= Pan-cytokeratin showing epithelium. **(D)** Col IV=Collagen IV showing basement membrane. **(E)** Ki-67 showing proliferative cells. **(F)** α -SMA = α -smooth muscle actin showing smooth muscle cells or myofibroblasts. Scale bar **(B–F)** 50 μm.

Grafts with ADM and GBA had a similar thickness to the native porcine vagina, whereas the grafts with PLGA and polyglactin mesh had a thickness of approximately a fifth of the native vagina (Fig. 3A). All grafts were covered with multi-layered epithelium on one side, closely mimicking the native vaginal epithelium and with positive staining for cytokeratin (Fig. 3B&C). Only grafts with polyglactin mesh had a clear and continuous basement membrane formation below the epithelium (Fig. 3D). Proliferative cells were abundant and present next to the basal parts of the epithelium on the native vagina and on all grafts except for the PLGA (Fig. 3E). Smooth muscle cells or myofibroblasts were present on the opposite graft side on ADM, GBA and polyglactin mesh grafts, and in the latter, the muscle cells were penetrating deep into the collagen and were seen covering the central polyglactin mesh fibres (Fig. 3F). Smooth muscle myosin −1 (SMMS-1), a major component of the contractile apparatus, showed a stronger signal in PLGA and polyglactin grafts (Supplementary Fig. 1).

### 2.4 PLATE grafts from other hollow organs

**Fig. 4:**
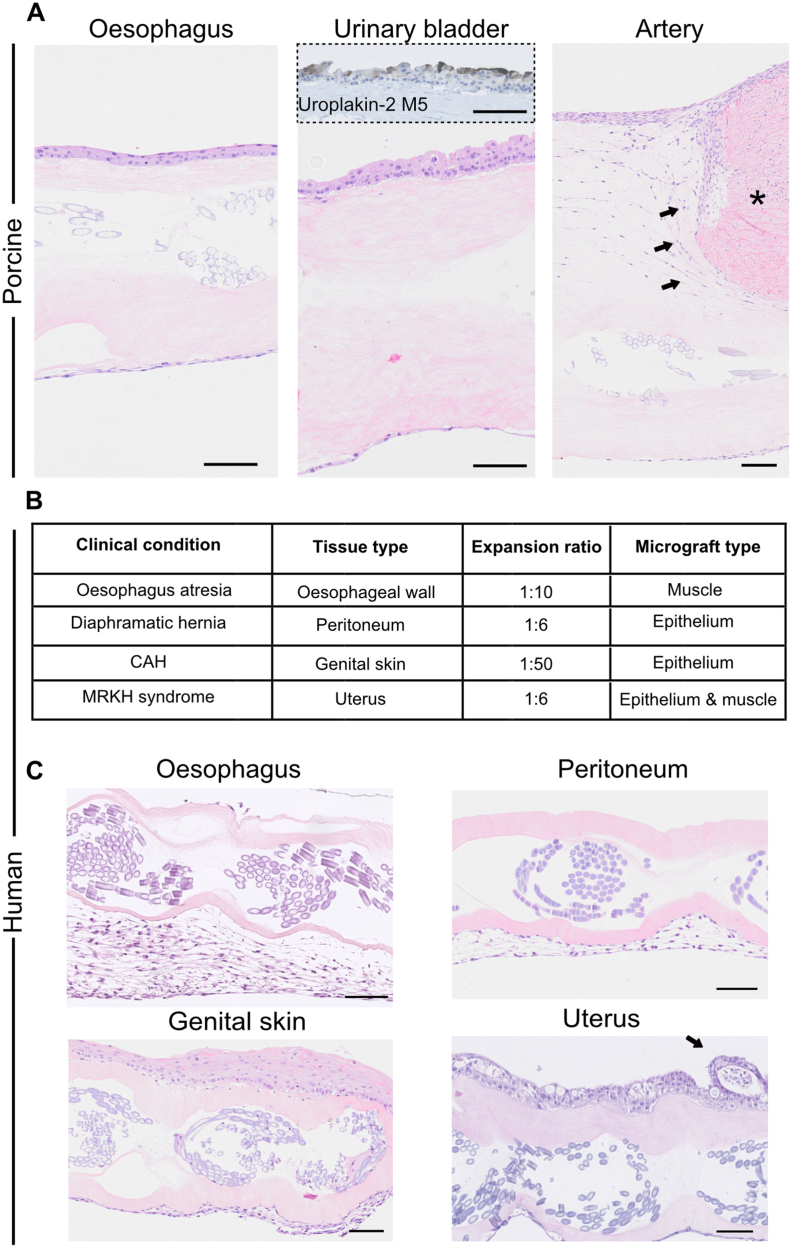
PLATE grafts from other porcine or human organs. (A) Porcine PLATE grafts after 4 weeks of *in vitro* tissue culture with epithelium on one side and muscle cells or fibroblasts on the opposite side from oesophagus (left), urinary bladder (middle) and from the aorta artery (right). Uroplakin-2 M5 is a marker for urothelial differentiation. Asterix = micrograft. Arrows show deep cellular migration from a micrograft into the collagen. **(B)** Table listing the origin of the human PLATE grafts shown in part C, and types of micrografts used. CAH = congenital adrenal hypoplasia **(C)** Human PLATE grafts after 4 weeks of *in vitro* tissue culture showing oesophagus, peritoneum, genital skin, and uterus. Arrow points at a suspected uterine gland. All H&E apart from the Uroplakin-2 insert, scale bars 100 μm.

We expanded the range of porcine organs and created PLATE grafts using the oesophagus, urinary bladder, and aorta. After four weeks, the oesophagus PLATE grafts displayed a multi-layered epithelium with non-keratinizing stratification, matching the oesophageal phenotype. On the opposite side, a thin layer of cells likely of smooth muscle or fibroblast origin was present. The urinary bladder PLATE grafts exhibited a continuous transitional epithelium with umbrella cell morphology and displayed a positive signal for uroplakin-2, a marker of terminal urothelial differentiation. On the opposite side, a thin layer of cells with smooth muscle morphology was present. The aorta grafts were colonized with cells containing elongated nuclei, likely of muscle or myofibroblast origin. A large tissue particle was compressed into the collagen part of the aorta graft, demonstrating cellular migration from the micrograft into the surrounding collagen (Fig. 4A).

### 2.5 PLATE grafts using human tissue samples

To bring the method one step closer to translation, we tested the PLATE graft technique using tissue samples from paediatric patients undergoing malformation surgery for various clinically relevant conditions (Fig. 4B). Small samples of the oesophagus wall from two patients with oesophageal atresia were used for PLATE graft fabrication at a 1:10 expansion ratio (the tissue sample was minced and distributed to a PLATE graft 10 times the original size of the tissue sample). After four weeks of *in vitro* culture, a thick layer of cells with muscle or myofibroblast morphology was observed on the seeded side.

A piece of hernia sac from a patient with diaphragmatic hernia was used for PLATE graft fabrication with a single layer of micrografts. After four weeks, a patent layer of cells displaying peritoneal morphology was observed. The largest expansion ratio of 1:50 was reached with a PLATE graft made from the genital skin of a young patient with congenital adrenal hyperplasia (CAH). After four weeks, a thick epithelial layer was observed on one side, along with cells displaying connective tissue morphology. These cells lined the polyglactin mesh fibers in the middle of the graft, despite micrografts having only been added to one side of the graft. A patient with Mayer-Rokitansky-Küster-Hauser (MRKH) syndrome had remnant uterine tissue removed, and endometrial and myometrium tissue from this patient was used for PLATE graft fabrication at a 1:6 expansion ratio. After *in vitro* culture, two distinct cellular colonies were present on either side of the graft. The top layer displayed columnar epithelium with ciliated and nonciliated secretory cells, as well as a potential gland formation, suggestive of uterine endometrium (Arrow, Fig. 4C).

### 2.6 Using PLATE grafts for vaginal reconstructive surgery in rabbits

**Fig. 5:**
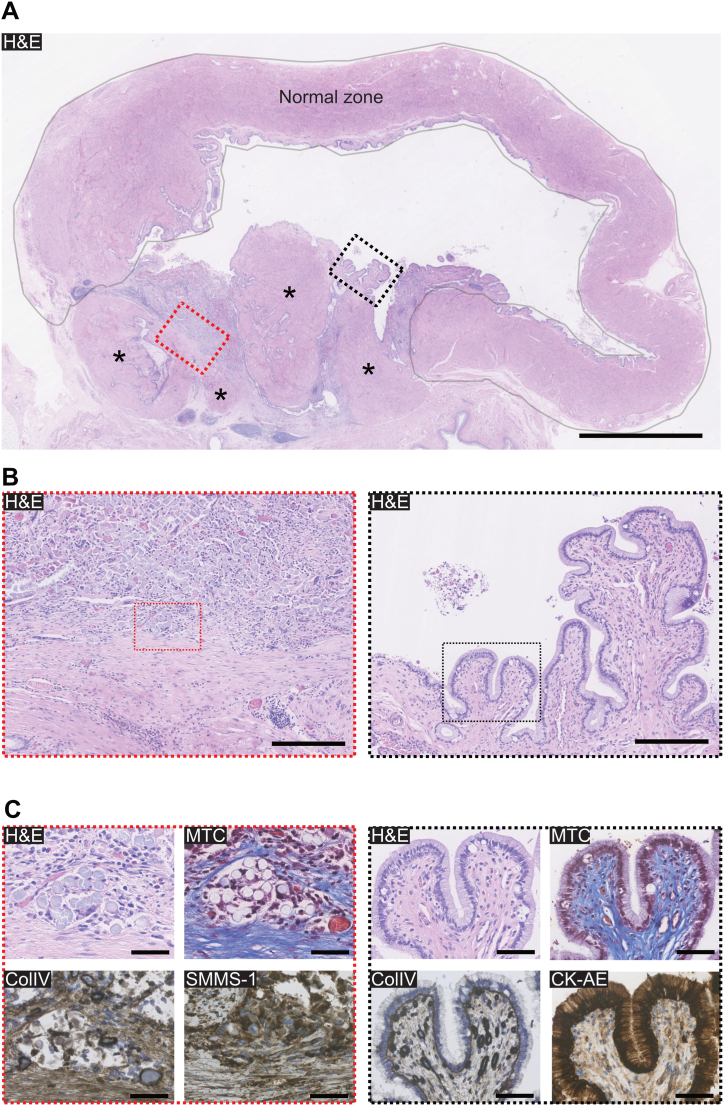
**PLATE grafts for vaginal reconstructive surgery in rabbits. (A)** Cross-section of the rabbit vagina four weeks after surgery showing the normal vaginal zone (enclosed area) and the PLATE graft zone located between the normal vagina borders. The red dotted square is a representative region of interest (ROI) from the central part of the graft zone, and the black dotted square is a representative ROI from the luminal part of the graft zone. Asterixis illustrate remnant micrografts. H&E, scale bar 2,5 mm. **(B)** Red dotted ROI (left) shows a densely populated stroma with cells around hydrolysing polyglactin mesh fibers (top half) or collagen (bottom half). Black dotted ROI (right) shows luminal neo-epithelium. H&E scale bars 250 μm. **(C)** Red ROI analysed with special stains to show polyglactin mesh fibers surrounded by cells of mixed phenotypes (SMMS-1 and H&E), a neovascular structure with erythrocytes (MTC) and basement membrane formation (ColIV). Black ROI shows a fully differentiated vaginal mucosa (H&E) with luminal epithelium (CK-AE), connective tissue (MTC) and a continuous basement membrane around the epithelial lining and capillaries (ColIV) scale bars 50 μm. (For interpretation of the references to colour in this figure legend, the reader is referred to the Web version of this article.)

During a single-stage surgery, vaginal PLATE grafts were successfully created and inserted into two anesthetized rabbits. The animals experienced no postoperative complications and were maintained for either two (n = 1) or four weeks (n = 1). Post-termination autopsies revealed well-integrated, patent grafts with no macroscopic signs of rejection or necrosis. Histological analysis at the four-week mark revealed a well-integrated PLATE graft zone without any exposed collagen, covering approximately one-third of the luminal surface. The graft zone displayed histological features consistent with the native vagina, including abundant cellular infiltrations in both the luminal and stromal regions. Four remnant micrografts were also identified (Fig. 5A).

The luminal mucosa within the graft zone was well-developed, featuring a continuous epithelium and an underlying basement membrane, supported by a bed of capillaries and connective tissue (Fig. 5B and C, right panels). In the deeper regions of the graft zone, hydrolysing polyglactin mesh fibers and collagen layers were interspersed with cellular infiltrates of various phenotypes, including smooth muscle cells that lacked bundle formation (Fig. 5B and C, left panels).

## 3 Discussion

The PLATE graft methodology was developed to explore the concept of *in vivo* tissue engineering. The aims of using the body as a bioreactor, only using low-cost materials, and finishing the procedure as a single surgery were all achieved. The hypothesis that the right biomaterial can provide the necessary biomechanical support in the short run, while providing the substrate for organised long-term graft cellularization in-vivo, seemed plausible.

The rationale of using micrografts for autologous tissue expansion was firmly established by Meek in 1958 when he used it for skin burn patients [18]. By recognising that during normal wound healing, epithelial cells migrate from the edges of the healthy tissue borders onto the wounded site, Meek improved wound healing by distributing minced tissue particles over the wounds, thereby increasing the graft surface area. Since, micrografts in various sizes and consistencies have been used for wound healing [19]. This basic principle has also been used for other types of organs in tissue engineering research, and our group has previously demonstrated how minced skin or bladder tissue particles can be distributed and glued directly onto silicone or latex tubes, thereby forming viable *in vivo* conduits [[20], [21], [22]].

In the interest of providing a better anchor for cellular attachment and migration, and making the constructs more robust, our method has since evolved. The tissue particles were placed onto a collagen hydrogel combined with a strong and biocompatible biomaterial. When this hybrid multi-layered construct was plastically compressed, all the components were fused together while the liquid component of the collagen hydrogel was removed, forming a surgically mendable graft. This concept has previously been tested using biomaterials made of poly-Ɛ-caprolactone (PCL) and poly(lactic-co-glycolic acid) (PLGA) in grafts intended for bladder urothelium expansion [23,24]. Our team has also previously surgically inserted plastic compressed grafts with minced epithelium and PCL underneath the skin in rats, as a preliminary safety trial, exploring the concept of autologous micrografting *in vivo* [25]. In those studies, only epithelium particles where implanted and none of the biomaterials used were commercially available. In a recent systematic review on vaginal tissue engineering, the combination of extracellular matrix-based biomaterials and autologous vaginal cells showed preferential properties, and our method draws on both of these concepts by utilizing both collagen type I gel, and minced autologous vaginal tissue particles [26].

The core biomaterial is important as a mechanical support for surgical tissue handling; moving to the surgical site and suturing the graft. From our present comparative studies, it was evident, that polyglactin mesh was superior to the three other biomaterials in terms of surgical handling, because of the thin and flexible design that allowed for easy manipulation and suturing. The perforated structure of the polyglactin mesh enabled the collagen to cross-link through the biomaterial, which made grafts robust and prevented collagen delamination. *In vitro* tissue regeneration was also superior with polyglactin mesh, both in terms of epithelial thickness, proliferation, basement membrane formation and also in terms of muscle cell proliferation and invasion into the deeper layers of the graft.

Biomechanically, polyglactin mesh was also superior to the other biomaterials in both strain and stress tolerance, and was the closest overall mimic of native porcine tissues. Knitted constructs are known as proper scaffolds for regeneration of load-baring tissues [27,28], and within this field of mechanobiology, it has been shown that stiffness of a biomaterial is known to give cellular state feedback, which can determine the fate of mesenchymal cell differentiation and is required to produce gene expression signatures that mimic the target tissues [29,30]. However, the force exerted on an organ and the required physiological functions can vary over time. The uterus, for example, is an organ that undergoes vast structural changes during pregnancy and can withstand large biomechanical forces during birth. It could withstand the least amount of internal force of all the organs and biomaterials tested. Had we used the uterus of a pregnant sow, the results would likely differ.

The biomechanical performance of the GBA, PLGA and polyglactin mesh grafts underwent a reduction after three weeks of *in vitro* culture (Fig. 2A&B). For this reason, polyglactin may not be superior for all applications. From another study, we know that the polyglactin mesh retains most of its biomechanical integrity after two weeks, so the biomechanical decay likely occurs between two and three week mark. We also know that it is stronger and less stretchable in the wale knit direction, unlike the coarse knit direction explored in the biomechanical tests presented here [31]. Therefore, for the grafts to effectively compensate for any biomechanical discrepancies that may arise after three weeks, it is crucial that they are thoroughly integrated into the host organism within that time-frame. The permeability assays indicated that three weeks of incubation reduced the permeability of the grafts, which is a likely attribute from the cellular barrier present on polyglactin mesh grafts at this time point in Fig. 3D. Moreover, in the *in vivo* pilot study where we used polyglactin mesh PLATE grafts, none of the animals showed any signs of graft failure at any point. The histology after four weeks showed a multitude of cells present within the full thickness of the graft site including the central regions where no cells were present under *in vitro* tissue culture conditions, indicating that the tissue regeneration is superior *in vivo* than *in vitro*, albeit less organised. This *in vivo* regenerative response is hypothesised to generate enough loadbearing tissue to alleviate the reduced biomechanical support expected from the polyglactin mesh appearing after three weeks, but this needs to be further assessed in *in vivo* functionality studies.

The polyglactin mesh used in this study has primarily been used for hernia repair, but is also being used as a biomaterial for a commercially available tissue-engineered product, where allogenic fibroblasts are cultured onto the mesh before being used on chronic wounds [32]. This supports that polyglactin mesh is a suitable biomaterial for translational tissue engineering.

Expansion ratios determine the distance between the micrograft islands and the distance for epithelialisation to transverse. They are calculated by dividing the area of the donated tissue by the area of the wound that needs to be covered by the graft. Ratios ranging from 1:6 to 1:150 have been presented in the literature, and for this study we also used different expansion rations depending on the organ and tissue source [19]. When using tissue samples from paediatric patients undergoing surgery, we could only use resected tissue that would otherwise be discarded, which often limited the quantity and useability. For each of the two oesophageal atresia patients, for example, a corner of approximately 2 × 2 mm was trimmed during the anastomosis closure to ensure a tight fit, but little or no epithelial lining was included in the resections. This meant that we could only engineer one-sided smooth muscle PLATE grafts. We had to push the expansion ratio boundaries for the genital skin PLATE graft (1:50). Despite the large ratio, we saw confluent layers of tissue after four weeks of culture, suggesting that the expansion ratio perhaps could be increased successfully.

In a Lancet commission on stem cells and regenerative medicine, it was recommended that research into making cell and gene therapies cost-effective and scalable should be a priority and that more evidence is needed for the cost-effectiveness of regenerative medicine [33]. The current variable cost of manufacturing a PLATE graft of 3 × 2 cm is approximately €20 EUR per graft which is cheaper than the typical price tag of a tissue cultured organ or a transplanted organ, not to mention the saved expenses from performing only one surgery. The reusable kit is very simple and cheap to manufacture and can be made in other sizes according to the desired applications. Technical experience with tissue dissection and mincing is required, and training is needed for surgeons to fabricate grafts with consistent results. Submerging the micrograft/polyglactin construct in collagen gel solution, transferring to a heating cabinet, and compressing, are all achievable within the confines of a normal surgical theatre, with little prior training. However, the general methodology presented here needs to be tailored for the specific hollow organ in question prior to clinical translation, and more experiments are needed to determine the optimal graft modifications needed. Also, even though the autologous tissue is removed from the patient and transplanted back into the patient during a single surgical procedure, the methodology would need to be approved by the governing health authorities prior to clinical use. To better democratise tissue engineering technology and prevent inequality in health, there is currently no patent or patent application hindering the use and replication of the method.

A previous animal study has suggested that the largest defect that an acellular graft can repair without fibrosis formation, appears to be approximately 1 cm^2^ [34], and another study showed a 60 % success rate for long-term urethral regeneration after inserting a 2 cm long acellular collagen urethral graft in a rabbit model [35]. To ensure that a critical size defect was created in our model, we replaced a 6 cm^2^ section of the vagina with a PLATE graft. At four weeks we saw regeneration across all the areas of the graft, including the central zones that are hard to reach from the adjacent regions. The pilot experiment with only two animals cannot determine if the cells have migrated or proliferated from the autologous micrografts or if the micrografts merely exert a paracrine function on other cells. To answer this question, a follow-up *in vivo* study comparing the regeneration of grafts with and without tissue particles would be needed.

There are limitations to the current PLATE graft methodology. Firstly, we used rat tail collagen type I, which is not yet approved for clinical use in humans. Existing today, however, are bovine, porcine and human collagen products, and even a recombinant human type I collagen, grown in tobacco plants, which has already been used in clinical trials without any systemic or local adverse effects [36,37]. Future studies would be needed to confirm the applicability of these alternative compounds. Secondly, the tissue regeneration we saw after four weeks *in vivo* was abundant, but less organised than in the native vagina. The *in vitro* culture conditions were static, and the regeneration would likely have been improved had we used more dynamic culture systems. Future *in vivo* studies need to be of adequate size, unlike the underpowered pilot experiments reported here. Indeed, more *in vivo* studies, comparing PLATE grafts with and without tissue particles from a histological, radiological, micromolecular and functional point of view, for long-term time points are warranted in both small and large animal models, in order to better our understanding of how PLATE grafts function *in vivo.*

## 4 Conclusions

PLATE grafts represent a different approach to tissue engineering that circumvents the need for custom bioreactors, multiple surgeries, GMP facilities, and round-the-clock laboratory staff. The method offers a low-cost alternative to traditional tissue expansion techniques and has clinical translational potential. While this paper tests the regenerative performance of PLATE grafts *in vitro* using porcine and human tissues, as well as *in vivo* in a small rabbit pilot study, additional experiments are needed to address all outstanding questions before PLATE grafts can be explored in human candidates.

## 5 Materials and methods

### 5.1 Study design

The objective of this study was to design and test a graft, that could be used for surgical reconstruction of hollow organs without many of the drawbacks from conventional tissue engineering. We first aimed at selecting the right biomaterial, and therefore tested four types of resorbable biomaterials. We tested both commercially available and custom-made, biological and synthetic, tightly woven and loosely knitted biomaterials with short and long resorption times, to cover a vast range of biomaterials. We assessed the biomaterials histologically and immunohistochemically with a qualitative tissue regeneration assessment after 3 or 4 weeks, which were timepoints determined prior to experimental commencement. Histological data using porcine tissue was generated in triplicates, one from each animal. Histological data from human tissue was only repeated once or twice due to limited availability of tissue. Biomechanical performance was assessed on fresh porcine organs in triplicates from three different pigs (n = 9 per condition) and in triplicates for each biomaterial (n = 3 per condition). Permeability assays were performed in two biological replicates with three technical replicates (n = 6 pr condition and timepoint). We used PLATE grafts for vaginal reconstruction in a live animal model with the aim to explore the single-stage surgery concept and test the *in vivo* response, and more details about the design of the animal study including ARRIVE guidelines are mentioned below.

### 5.2 Ethics

This study was reviewed and approved by the Capital Regional Ethical Committee (VEK) with the approval number: H-20084445. The animal study was approved by the national inspectorate for animal experimentation and was carried out in accordance with the EU directive 2010/63/EU for animal experiments with the approval number: 2021-15-0201-00982.

All participants/patients (or their proxies/legal guardians) provided informed consent to participate in the study.

All participants/patients (or their proxies/legal guardians) provided informed consent for the publication of their anonymised case details and images.

### 5.3 Tissue preparation

For fabrication of a 3 × 2 cm graft, a donor tissue specimen of approximately 1 cm^2^ was harvested to allow for a 1:6 expansion ratio. The epithelium and smooth muscle layers were separated by surgical dissection using basic micro-surgical instruments. The thickness of the epithelial layer was determined by the native epithelium (0,5-1 mm), and the thickness of the smooth muscle autograft was trimmed to approximately 1 mm using round edge scissors. If no muscle layer was visible macroscopically, which was often the case with the smaller human tissue samples, the deeper part of the connective tissue layer was used instead. Then, the epithelial and muscle layers were minced separately into approximately 1,5 x 1,5 × 1 mm (length, width, and thickness) cubes with a round edge scalpel (Type 21, Swann-Morton, UK) (Fig. 1B). To reach consistent micrograft dimensions, we made all the longitudinal cuts first, and made orthogonal cuts in the other direction afterward. To avoid dehydration, a 200 μL of DMEM 1X (Gibco, Thermo Scientific, USA) was added on the piles of micrografts.

### 5.4 Graft fabrication for *in vitro* culture

Collagen gel preparation was performed as previously described [38]. In brief, a solution was prepared by mixing 6 ml of ice-cold rat-tail collagen type I (2 mg/mL protein in 0.6 % acetic acid, First Link, UK) with 1 mL of 10X MEM (Gibco, Thermo Scientific, USA), approximately 1,25 mL of NaOH (1 mol/L, Supelco, USA) added dropwise to allow a pH of 7. 2, and 1 mL of 1x DMEM (Gibco, Thermo Scientific, USA). 3 ml of this solution was poured into a rectangular mould of 3x2x1 cm (length x width x depth) and allowed to polymerise at 37 °C for 5 min. Once the base collagen solution polymerised, a 2 × 3 cm piece of a biomaterial was placed on top, and another 3 mL of the collagen solution was added. Gel polymerisation was allowed to proceed for the top collagen layer for another 10 min at 37 °C. To get tissue particles on either side of the graft, we first distributed the epithelium micrografts particles on top of the solidified gel using forceps. To get muscle or connective tissue particles on the underside of the gel construct, we distributed muscle or connective tissue micrografts onto monofilament polyamide mesh (pore size 20 μm Schwegmann, Germany) before placing the gel construct directly on top of the particles. Below the polyamide mesh, we placed a blotting set with a perforated steel plate and a 2 cm pile of sterile gauze (10 × 10cm, Selefa, Finland). On top of the construct, we placed another piece of polyamide mesh. The final step to form the PLATE graft was obtained by removing water molecules from the gel by placing a steel plate and a weight (approximately weighing 120 g) on top of the gel for 5 min (Fig. 1B). The steel cast and plates were custom-made by a hospital technician and were autoclaved prior to each use.

### 5.5 Graft *in vitro* culture

The grafts were sectioned into four smaller squares of 1 × 1.5 cm, and placed into individual wells in 12-well tissue culture plates (Cellstar, Greiner, Austria) and cultured submerged in keratinocyte growth medium as previously described [22]. The medium was changed every two to three days.

### 5.6 Biomaterial source and preparation

Knitted polyglactin 910 mesh (Vicryl® mesh, Ethicon, USA) was referred to as polyglactin mesh. Gore® BIO-A® Tissue Reinforcement 67 % polyglycolic acid & 33 % trimethylene carbonate (W.L Gore & Associates, Inc. USA) was referred to as GBA. ARTIA Reconstructive Tissue Matrix (Lifecell Corporation, USA) was referred to as ADM*.* There three biomaterials were all commercially available off-the-shelf products. Electrospun poly(lactic-co-glycolide), referred to as PLGA, with a lactide:glycolide ratio of 75:25 was custom-made in accordance with a previous publication [24]. Briefly summarised, a 16 % (w/v) solution of PLGA in Chloroform/DMF (90/10 v/v) was stirred overnight and used for electrospinning at a voltage of 16 kV using a 21G blunt needle attached to a 1 mL syringe. The collector surface was covered with a plastic mesh to pattern the PLGA electrospun sheet with texture squares to avoid delamination between the PLGA sheet and the compressed collagen.

All biomaterials were sectioned into 2 × 3 cm squares prior to use under sterile conditions. The ADM was further modified by making superficial scalpel scratches in all directions and small scalpel punctures in the longitudinal direction to further texturize the surface and reduce delamination.

### 5.7 Porcine tissue collection

Porcine organs were collected from newly-euthanized female Danish landrace pigs of around 35 kg, that had been used for unrelated surgical training procedures (additional ethical permission was not needed in euthanized pigs). After the administration of a lethal dose of pentobarbital to the anesthetised pig, all the abdominal organs were surgically resected via a midline incision in the abdomen. The oesophagus and trachea were harvested through a midline anterior cervical incision, and the oesophagus tissue was harvested from the middle segment of the organ, where no skeletal muscle cells were expected to be present. The pubic symphysis was opened to better access the urethra, vagina and rectum.

All organ segments intended for biomechanical tests were placed into 50 mL centrifugation tubes with sterile PBS (Gibco, Thermo Fischer Scientific, USA) on ice for up to 8 h. To procure aseptic handling, organ segments for tissue culture were dipped into 70 % ethanol for 2 s, air dried briefly, and placed into sterile centrifugation tubes with sterile PBS supplemented with 1 % penicillin and streptomycin and 2.5 μg/ml amphotericin B, the latter only being added for the vaginal samples.

### 5.8 Human tissue collection

Surgical tissues from paediatric patients were collected at the operating theatre and kept moist in sterile saline in centrifugation tubes. We only used resected tissue that would otherwise be discarded and after preoperative signed informed consent.

### 5.9 Biomechanical tests

Uniaxial tensile tests were performed using a Texture Analyzer (Stable Micro Systems, UK) at strain speeds of 10 mm/min until rupture. The ultimate tensile strength and the corresponding strain at that point were used for our analysis. Using forceps, the tissue was suspended between the clamps with a 20 mm gap between the jaws, thus giving a test area 1 cm^2^ (20 mm × 5 mm). All grafts and native tissues were cut into uniform sizes of 50 mm × 5 mm, and were reheated to 37 °C and covered in liquid by submerging the tissues in heated 1X DMEM prior to analysis. All biomechanical tests on native tissues were repeated in three different animals in triplicates, yielding n = 9 samples per condition. Biomechanical tests on grafts made from the different biomaterials were performed in triplicates yielding n = 3 samples per condition. Fresh grafts did not contain any tissue particles and were kept in 1X DMEM for a few hours prior to the tensile tests. Long-term biomechanical properties were assessed on porcine vaginal tissue grafts cultured in incubators for 21 days (n = 3 per condition). Polyglactin mesh grafts were tested in the course knit direction.

### 5.10 Permeability assays

We used two unjacketed Franz diffusion cells with a 1 mL donation chamber volume, and a 5 ml receptor volume (SES GmbH Analyse Systeme, Germany). Each chamber was separated by 1 cm^2^ of a porcine vaginal PLATE graft as a membrane between the two compartments. Bovine serum albumin (Sigma-Aldrich, USA) at a concentration of 12 mg/mL was added to the donation chamber, and Milli Q ultrapurified water was added to the collection chamber. Albumin is the most abundant circulating protein and was chosen for its large size and spectrophotometric absorbance properties. Assays were performed under physiological conditions inside an incubator at 37 °C. 20 μL samples were collected from the collection chamber at 30, 60, 120, 180, 240 and 300 min and were analysed with a UV–Vis spectrophotometer (NanoDrop™ 2000 Thermo Fisher, USA) at 278 nm to quantify the albumin concentration. A standard albumin curve was used to accurately quantify the concentration of albumin from the absorbance levels.

### 5.11 Histology

Native organs and *in-vitro* cultured grafts were cut into three to four strips and put into cassettes using the Tissue-Tek® Paraform® sectionable cassette system (Sakura, USA) and placed in 4 % formaldehyde (VWR chemicals, USA) for at least 24 h. Rabbit vaginas from the *in vivo* animal study were resected along with the bladder and fixed in 4 % formaldehyde for at least 24 h. The organs were then sectioned transversely into 5 mm sections including both the vaginal and bladder lumina. They were placed into cassettes, underwent tissue dehydration, paraffin embedding, and microtome sectioned at 3 μm. Sections were plated and stained with either haematoxylin & eosin (H&E), Masson's trichrome (MTC) or immunohistochemistry stains, performed in accordance with routine protocols for clinical practise. Immunohistochemical stains were counterstained with haematoxylin and validated against positive controls on the same slide by a certified pathologist. We used pan-cytokeratin CK-AE (Clone AE1/AE3, ID: GA053, DAKO Agilent, USA), Collagen IV (clone CIV 22, ID: 760–2632, Roche, Switzerland), Ki 67 (clone MiB-1, ID: GA626, Agilent, USA) alpha smooth muscle actin (clone 1A4, Agilent, GA611), Smooth muscle myosin −1 SMMS-1 (Mouse monoclonal, ordering code: 05268133001, Roche, Switzerland) and Uroplakin II (Clone BC21, ACI3051C, Biocare, The Netherlands).

antibodies. Slides were scanned using a slide scanner (Visiopharm Oncotopix scan, Hamamatsu, Japan).

### 5.12 Scanning electron microscopy

Biomaterials were fixed in 4 % formaldehyde overnight and were dehydrated with increasing concentrations of ethanol (30 %, 50 %, 70 %, 90 %, 100 % and 100 % again) for 10 min each. The biomaterials were then treated with hexamethyldisilazane (Sigma-Aldrich, USA) overnight for further water extraction. Finally, they were mounted on aluminium stubs using double sided carbon tape (Agar Scientific, UK), and imaged using a tabletop scanning electron microscope (TM3030Plus, Hitachi Hightech, Japan) at various magnifications.

### 5.13 In vivo experimentation and animal model

Two female White New Zealand rabbits (Charles River, France) of approximately 18 weeks and 3 kg were used for an exploratory *in vivo* pilot experiment for vaginal reconstruction. Anesthesia was induced with dexmedetomidine (0,02 g/kg IM) + midazolam (0,2 mg/kg IM) + buprenorphine (0,05 mg/kg IM) and 5 min later ketamine (6.5 mg/kg IM), and was sustained with sevoflurane (2.5 % in 35 % O2 inhalation) and fentanyl (5 μg/kg/hour IV). Rabbits received postoperative analgesia with a local injection of bupivacaine (2 mg/kg SC) once, buprenorphine (0.01–0.05 mg/kg/sc) two to three times a day for three days, and metacam (1 mg/kg oral suspension) for four days, and antibiotics sulfadioxin/trimetrioprim (0.1 ml/kg SC) one day prior to surgery and four days post-surgery.

During a single-stage surgery, a 3 × 2 cm anterior segment of the rabbit's vagina was excised, leaving behind a native strip of tissue connecting the two ends of the vaginal tube. A fifth of the excised tissue was then used for PLATE graft fabrication with epithelial particles on one side and mixed connective and muscle tissue particles on the other, at a 1:5 expansion ratio. We used a larger steel mould of 4 × 6 cm, 24 mL of the collagen solution, and a piece of polyglactin mesh of 3 × 2 cm to make grafts. The collagen was polymerised inside in a transportable oven set at 37 °C and placed inside the operating theatre. Excess collagen around the edges of the PLATE grafts was trimmed with a scalpel prior to graft insertion *in-vivo*. Grafts were inserted with running monofilament sutures, and the vagina was re-attached to the bladder with three interrupted sutures adjacent to the graft. Graft fabrication took 30 min, and was performed by the surgeon inside the operating theatre. A foley-type catheter was placed inside the vagina and fixed to the vaginal introitus using resorbable sutures. Animals were kept alive for 2 weeks (n = 1) or four weeks (n = 1) before euthanasia with intracardial injection with pentobarbital (100 mg/kg) under anesthesia with ketamine (30 mg/kg IM) and xylazine (5 mg/kg IM). There was no randomisation, control group or blinding during this exploratory pilot experiment.

### 5.14 Statistical analysis

For the biomechanical tests, results were presented as mean values from each condition (n = 9 for biological samples and n = 3 for engineered grafts) and error bars represented standard deviations. For permeability assays, two biological conditions were analysed with three technical replicates, that were used for mean value calculations. Error bars represented the standard error of the mean (SEM). We used two-tailed student's t-tests for p-value calculations, where significance was defined as p < 0.05.

## Funding

This work was supported by the Birgitta and Carl-Axel Rydbeck's Research grant for Paediatric Research and the 10.13039/100009389Promobilia Foundation in Sweden, and the 10.13039/501100009708Novo Nordisk Foundation NNFSA170030576 in Denmark.

## Data materials availability

Data associated with the study has not been deposited into a publicly available repository. All non-patient data is available on request to the corresponding author.

## CRediT authorship contribution statement

**Oliver Willacy:** Writing – review & editing, Writing – original draft, Visualization, Project administration, Methodology, Investigation, Formal analysis, Data curation, Conceptualization. **Nikolai Juul:** Writing – review & editing, Writing – original draft, Methodology, Investigation, Data curation, Conceptualization. **Loai Taouzlak:** Software, Methodology, Formal analysis. **Clara I. Chamorro:** Writing – review & editing, Writing – original draft, Supervision, Methodology, Conceptualization. **Fatemeh Ajallouiean:** Writing – review & editing, Writing – original draft, Validation, Supervision, Resources, Methodology, Data curation. **Magdalena Fossum:** Writing – review & editing, Writing – original draft, Validation, Supervision, Resources, Project administration, Methodology, Investigation, Funding acquisition, Conceptualization.

## Declaration of competing interest

The authors have no competing interests.

## Figures and Tables

**figs1:**